# Cyclodextrins as building blocks for new materials

**DOI:** 10.3762/bjoc.19.66

**Published:** 2023-06-19

**Authors:** Miriana Kfoury, Sophie Fourmentin

**Affiliations:** 1 Université du Littoral Côte d'Opale, Unité de Chimie Environnementale et Interactions sur le Vivant (UCEIV, UR 4492), 59140 Dunkerque, France

**Keywords:** building blocks, cyclodextrins, new materials

The continuous increase in the number of publications carried out on cyclodextrins (CDs) and their market growth are clear evidence of the undying interest in these macrocycles discovered in 1891 [1]. It seems that these supramolecules still have not revealed all their secrets and are still stimulating curiosity in fundamental and applied research. CDs are the most studied supramolecular hosts. They provide the most extensive database on molecular recognition in the literature, with more than 100,000 publications [2]. CDs owe their success not only to the unique molecular structure [3], which allows them to act as host compounds, but also to their biodegradability [4], negligible toxicity, and excellent safety profile [5]. In addition, CDs offer a further step towards sustainability, making them suitable for a wide range of uses in various fields. Accordingly, the global market size of CDs is expected to grow to nearly US$ 390 million by 2027, registering consequently a compound annual growth rate of 5.5% from 2021. The CD market was not affected by the COVID-19 pandemic and had in fact experienced a strong increase. This was due to the favorable environment for the CD market created by the increasing demand for pharmaceuticals containing CDs. Three clinical trials have demonstrated the use of CDs in the treatment of COVID-19. Two of them use the sulfobutyl ether β-CD/remdesivir inclusion complex and the third applies the α-CD/sulforaphane inclusion complex called Sulforadex^®^ [6]. The approved Janssen vaccine against SARS-CoV-2 infection (Ad26.COV2.S) was also a milestone for CD research and has served as a large-scale safety test for 2-(hydroxypropyl)-β-CD (HP-β-CD). CDs have also been used to functionalize face mask textiles to block and inactivate bacteria and viruses [7].

Apart from the COVID-19 pandemic, CDs are well represented in the pharmaceutical market, in at least 130 marketed products [2]. Examples of the use of CDs in medicines are β-CD in cetirizine tablets and cisapride suppositories, γ-CD in a minoxidil solution, HP-β-CD in itraconazole antifungal, in intravenous and oral solutions, sulfobutyl ether β-CD in intravenous voriconazole antimycotic, and randomly methylated β-CD in a nasal spray for hormone replacement therapy with 17β-estradiol.

Yet, the current interest goes far beyond the simple use of CDs as a delivery system for therapeutic agents. Current research interests focus on the intrinsic activity of CDs as well as their derivatives and polymers. One of the biggest discoveries in this field is the observation that the solubilizer HP-β-CD is effective against Niemann–Pick type C disease (NPC) and is in phase 2b/3 clinical trials [8]. It has also been suggested that CDs may have beneficial effects on other neurodegenerative diseases, such as Alzheimer’s, Parkinson’s, and Huntington’s diseases. Due to their ability to extract cholesterol, treatment with CDs could reduce atherogenesis and atherosclerotic plaque size by solubilizing cholesterol crystals [9]. In addition, CDs can sequester cholesterol and lipids from viruses and envelopes and provide virucidal and bactericidal activity against a wide range of microorganisms [10]. Altogether, these findings highlight the ability of CDs to act as potential active pharmaceutical ingredients, which may influence the current regulatory framework for the use of CDs and further stimulate their market.

The exploration of the potential of inclusion complexation is not limited to the biomedical field. CDs are renewable and biodegradable materials that enable green and environmental biotechnologies for all applications [11]. The ability of CDs to act as solubilizers, stabilizers, permeation enhancers, cryoprotectors, sequestrants of toxic compounds, taste and odor maskers, coating materials of solid surfaces, and chiral receptors has been successfully explored in food, packaging, cosmetics, textiles, separation processes, environmental remediation, extraction, and catalysis [2,5,12].

Molecular encapsulation is not the only application area for these macrocyclic components at present. CDs are versatile molecules. Their 3D structure makes them exceptional building blocks for the design of innovative supramolecular architectures due to the differential reactivity of their alcohol functions. This allows regioselective chemical modification at either the primary or secondary rim [13]. As a result, these molecular hosts can be specifically linked either covalently or noncovalently to a wide variety of ligands. CDs are a significant part of almost all areas of science that require high performance with minimal environmental impact. They are involved in the construction of interlocked molecules (rotaxanes and catenanes), supramolecular polymers, artificial enzymes, hydrogels, metal–organic frameworks, supramolecular solvents, fibers, nanotubes, nanoparticles, and so on [14–17]. In addition to bearing a rigid skeleton, CDs act as versatile multitasking agents. They add value to these composites as their cavities remain generally available to accommodate active substances, or they work as supramolecular catalysts or molecular concealers.

Due to the fact that CDs are nontoxic to humans and to the environment and used to develop greener synthetic routes and strategies, CD-based materials are considered safe and environmentally friendly [18]. This includes, among other characteristics, that they are edible, biodegradable, ecological, and biocompatible. Thus, these supra-architectures have a multitude of uses in food, biomedicine, regenerative medicine, cosmetics, molecular electronics, polymer chemistry, gold recovery, gas absorption, depollution, biochemical material sciences, nanotechnology, self-healing materials, 3D printing, and so on [19–21].

We hope that you will enjoy consulting the articles in this thematic issue and that you will gain new insights to help advance CD-based materials, which are burgeoning on several fronts and are likely to continue to push the boundaries in all areas of applications and beyond. We are extremely grateful to all the researchers who contributed to this issue and thankful to the Editorial Team at the Beilstein-Institut for their kind assistance and support.

Miriana Kfoury and Sophie Fourmentin

Dunkerque, June 2023
